# Spirochaetes dominate the microbial community associated with the red coral *Corallium rubrum* on a broad geographic scale

**DOI:** 10.1038/srep27277

**Published:** 2016-06-06

**Authors:** Jeroen A. J. M. van de Water, Rémy Melkonian, Howard Junca, Christian R. Voolstra, Stéphanie Reynaud, Denis Allemand, Christine Ferrier-Pagès

**Affiliations:** 1 Centre Scientifique de Monaco, 8 Quai Antoine 1er, MC 98000, Monaco; 2 Microbiomas Foundation – Division of Ecogenomics and Holobionts, Chía, Colombia; 3 Red Sea Research Center, Biological & Environmental Sciences & Engineering Division, King Abdullah University of Science and Technology (KAUST), Thuwal, Saudi Arabia

## Abstract

Mass mortality events in populations of the iconic red coral *Corallium rubrum* have been related to seawater temperature anomalies that may have triggered microbial disease development. However, very little is known about the bacterial community associated with the red coral. We therefore aimed to provide insight into this species’ bacterial assemblages using Illumina MiSeq sequencing of 16S rRNA gene amplicons generated from samples collected at five locations distributed across the western Mediterranean Sea. Twelve bacterial species were found to be consistently associated with the red coral, forming a core microbiome that accounted for 94.6% of the overall bacterial community. This core microbiome was particularly dominated by bacteria of the orders Spirochaetales and Oceanospirillales, in particular the ME2 family. Bacteria belonging to these orders have been implicated in nutrient cycling, including nitrogen, carbon and sulfur. While Oceanospirillales are common symbionts of marine invertebrates, our results identify members of the Spirochaetales as other important dominant symbiotic bacterial associates within Anthozoans.

The economically valuable red coral *Corallium rubrum* is an important habitat-forming species that provides structural complexity to benthic communities in the Mediterranean Sea. While commercial over-exploitation for use in jewellery is one of the main threats, ocean acidification^1^ and elevated seawater temperatures^2^ may also pose a risk to *C. rubrum* populations. High seawater temperatures are known to cause changes within coral microbial communities resulting in a higher abundance of potential pathogens^3^,^4^, which may partially explain the mass mortality events of gorgonians documented throughout the Mediterranean Sea^2^,^5^. While two studies have provided initial efforts to identify bacteria associated with the red coral^6^,^7^, the composition of bacterial assemblages associated with this species *in situ* is still largely unknown.

Several studies have identified members of the order Oceanospirillales, in particular those from the genus *Endozoicomonas*, as major bacterial associates (up to 90%) of temperate gorgonians^8^,^9^,^10^,^11^, as well as other Anthozoans^12^,^13^,^14^ and marine invertebrates^15^,^16^. While this suggests *Endozoicomonas* spp. may be symbionts, as has been described for scleractinian corals^12^,^17^, it is unknown what the role of this genus is in the red coral^6^. Given the increasing pressures on *C. rubrum* populations, it is important to assess in detail the natural bacterial assemblages associated with *C. rubrum* as this may help to understand their potential function within holobiont health and provide a basis for understanding the causes of population declines. In this study, we therefore investigated the spatial diversity of the bacterial communities associated with *C. rubrum* colonies within the western Mediterranean Sea.

## Results and Discussion

**Figure 1:**
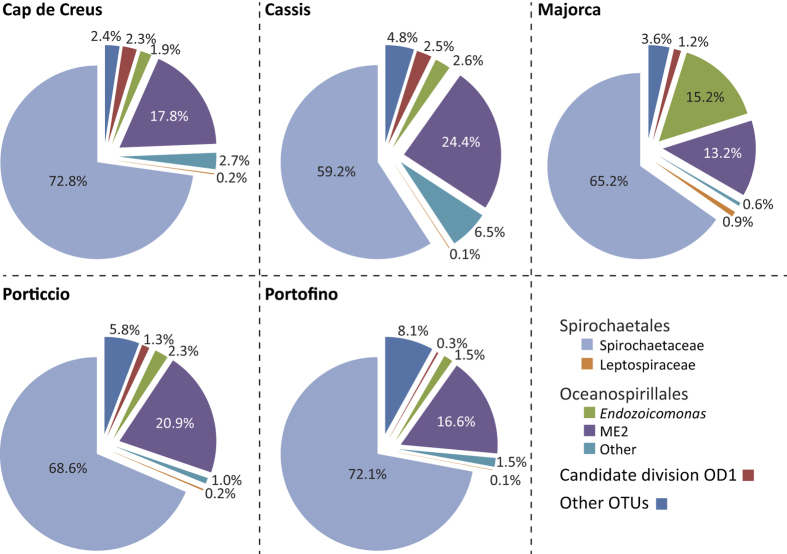
Overview of the composition of the microbiomes associated with *Corallium rubrum* showing relative spatial stability. The contribution of each taxon to the microbiome at each location is indicated in percentages (%).

**Figure 2:**
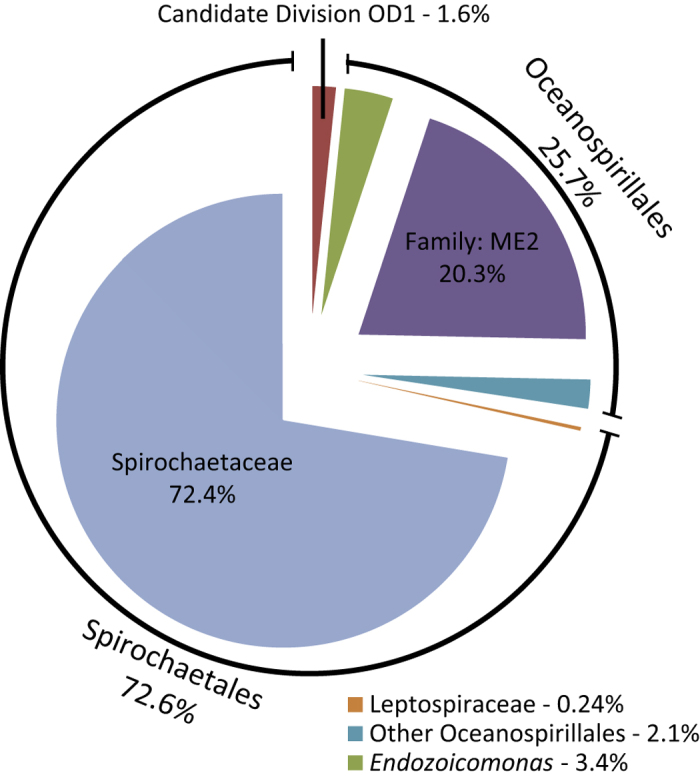
Overview of the composition of the core microbiome associated with *Corallium rubrum*. The contribution of each taxon to the core microbiome is indicated in percentages (%). All taxa are represented by a single Operational Taxonomic Unit (OTU), except Spirochaetaceae (4 OTUs) and Oceanospirillales from the family ME2 (4 OTUs).

Initially, we confirmed that microbial communities associated with *C. rubrum* were significantly different from bacterial communities in the seawater based on alpha diversity metrics (p < 0.0001; Table 1), bacterial species (classified as Operational Taxonomic Units (OTUs) with at least 97% similarity) present (p < 0.0001; Supplementary Fig. S1A,C) and community structure (p < 0.0001; Supplementary Fig. S1B,D), indicating host specificity. The microbial communities associated with the red coral were dominated by bacteria belonging to the orders of the Spirochaetales (68.0% ± 9.3) and Oceanospirillales (25.4% ± 7.7) at all locations (Fig. 1). The low evenness found within the communities (Table 1), despite a high number of OTUs (250 ± 20) present, suggests the dominance of only a few OTUs. Further analysis revealed that *C. rubrum* possesses a substantial and highly conserved core microbiome, which we define as the bacterial community consistently associated with *C. rubrum* (i.e. OTUs present in all samples regardless of location). The core microbiome of the red coral encompasses 94.6% (±2.65) of the entire microbial community (Supplementary Table S1) and is represented by 12 OTUs (Fig. 2, Supplementary Table S2). No differences in the microbial community associated with *C. rubrum* were observed between 4 of the 5 locations, showing that the relative abundances of these OTUs within the core microbiome were relatively stable on a spatial scale (Figure S2). However, red corals near the island of Majorca harboured significantly different microbiomes in comparison to the other locations (Figure S2). This dissimilarity in microbial community composition could largely (>80%) be attributed to changes in the abundances of members within the core microbiome (Supplementary Table S3), rather than a change in microbial community membership (Supplementary Fig. S3). Particularly, there was a shift towards a lower relative abundance of the generally most dominant Spirochaetales (KT964897) and Oceanospirillales (KT964893) OTUs, while the second most abundant Spirochaetales (KT964901) and Oceanospirillales (KT964903) OTUs increased in numbers (Fig. 1; Supplementary Table S3). Overall, this indicates that acclimation of the *C. rubrum*-associated microbial community to local conditions is largely regulated by restructuring of an otherwise stable core microbiome. Conversely, we found that, on a local scale, the red coral harboured communities of locally stable microbial associates (i.e. species present in all samples from a given location) of which the membership was distinct at each location (Supplementary Fig. S4). These spatial differences in membership could, however, primarily be attributed to numerous bacterial species that were either very low abundant or absent, as they did not contribute significantly to the dissimilarity in the overall microbial communities. While the role of these very low abundant locally stable associates is unknown, it may suggest local specificity possibly due to selection of the microbial community to local conditions by the red coral or the transient uptake of locally present bacterial species^18^. Taken together, the significant portion of the microbial community represented by the members of the core microbiome and the stability of the interactions between these bacterial species and *C. rubrum* show their importance to host health through strong associations.

We show that members of the order Spirochaetales are the predominant bacterial associates of the red coral, which is in stark contrast to the *Endozoicomonas*-dominated bacterial communities described in other Anthozoans^8^,^9^,^10^,^11^,^12^,^13^,^14^. While Spirochaetales harbours several species that cause disease in vertebrates, in some invertebrates, such as arthropods, molluscs and oligochaete worms, Spirochaetes are known abundant endosymbionts^19^,^20^,^21^. The Spirochaetes present in termite guts are the best studied and have been found to be involved in the breakdown of lignocellulose^20^ and nitrogen fixation^22^. In tropical corals, however, the role of these bacteria is unclear as they have been found at low abundance associated with both diseased^23^,^24^,^25^ and healthy corals^23^,^26^,^27^. In addition, several reports have found associations between Spirochaetes and the cold-water coral *Lophelia pertusa*^28^, the deep-sea bamboo coral *Isidella tentaculum*^29^ and the gorgonian *Thouarella superba*^30^, but Spirochaetes are only present at relatively low numbers in these organisms. Interestingly, unclassified sequences that closely match with our Spirochaetes OTUs (identity match of 96–100%; Supplementary Table S4), were recently observed at low abundance (5.3%) in the red coral^6^. Transient changes in gorgonian-associated microbiomes, as reported for *Paramuricea clavata*^9^, may explain the differences between these studies. However, it cannot be excluded that differences in sequencing methodologies and depth give rise to some of these discrepancies.

**Figure 3:**
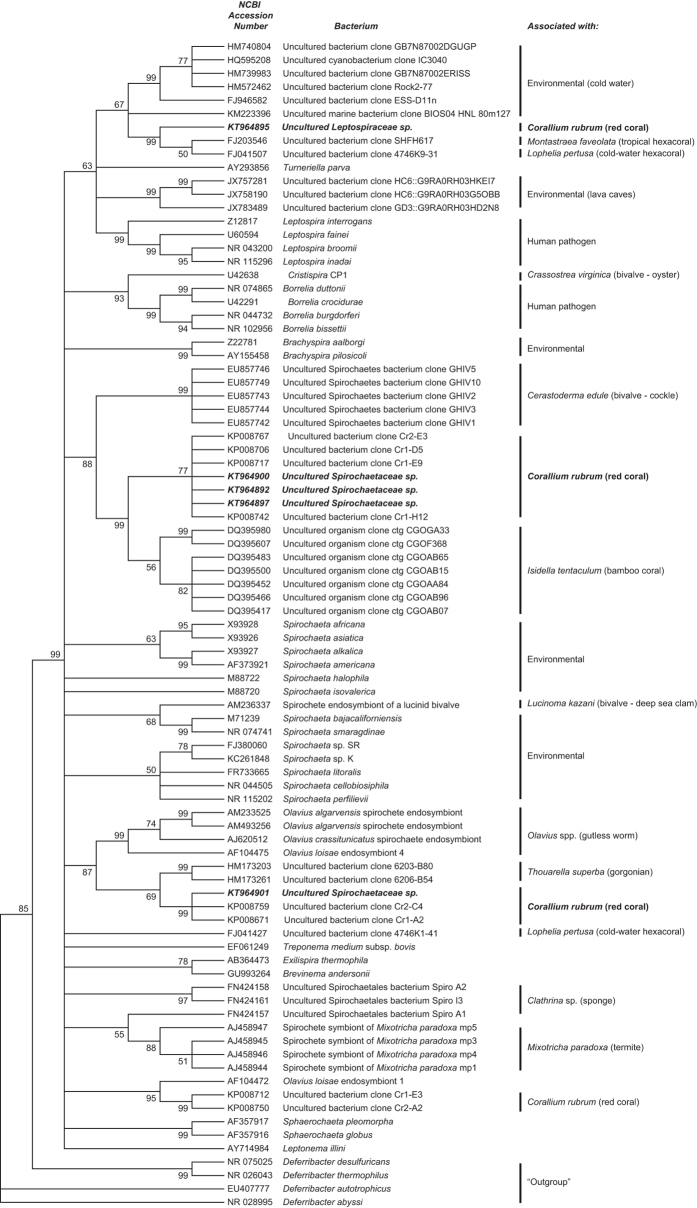
Phylogeny of Spirochaetes. A 16S ribosomal RNA reconstruction of the Spirochaetes created based on the maximum parsimony model. Percentages of 1000 bootstrap replicates are indicated next to the tree nodes if they are >50%. Sequences from the present study are in **bold** and *italic*. The tree is rooted using sequences from the phylum *Deferribacter* as the outgroup.

Phylogenetic analysis of the Spirochaetales OTUs observed in the red coral core microbiome in our study showed that four OTUs, potentially belonging to the family *Spirochaetaceae*, clustered primarily with clones previously isolated from *C. rubrum*^6^, *I. tentaculum*^29^ and *T. superba*^30^ (Fig. 3; Supplementary Table S4). The Leptospiraceae OTU clustered with bacterial clones retrieved from the hard corals *L. pertusa* and *Montastrea faveolata*^31^, and cold water environments (Fig. 3; Supplementary Table S4). While two Spirochaetaceae OTUs (KT964897, KT964901) were highly abundant, the other Spirochaetales core microbiome members were present at low numbers (Supplementary Table S2). Their consistent presence, however, suggests that they probably play an important role in host physiology^26^. While the functions of these bacteria in the red coral are unknown, symbiotic Spirochaetes have been shown to be involved in the fixation of nitrogen^22^ and carbon^32^ for use by the host and its associated microbial community. Consequently, the red coral-associated Spirochaetaceae may provide the host with similar symbiotic functions as *Symbiodinium* and nitrogen-fixing bacteria in tropical reef-building corals. Although Spirochaetales may be common associates of cold water gorgonian species, their particularly high abundance within the red coral’s core microbiome shows that *C. rubrum* has a distinct microbiome within the Anthozoans.

The other major group of bacterial associates of the red coral found in this study were bacteria belonging to the order of Oceanospirillales. In particular, OTUs belonging to the ME2 family made up a large portion of the core microbiome (20.3%), but the functions of bacteria from this taxon are currently unknown. Most Oceanospirillales OTUs identified in our study were closely related to those previously found in the red coral (Supplementary Fig. S5; Supplementary Table S4). In contrast to other Anthozoans, the role of *Endozoicomonas* spp. in *C. rubrum* (3.4% of the core microbiome) appeared to be relatively minor, but may still be highly relevant to host physiology^26^. These results emphasise the unique, but conserved microbiome associated with the red coral.

The highly spatially stable and unique association of the red coral with the members of its core microbiome raise questions about the acquisition of these microbial symbionts. As *C. rubrum* is a brooding species, it is likely that the red coral inherits its core microbiome through vertical transmission from the brooding parental colony to the planulae^33^, explaining the high geographic stability of the red coral microbiome. The three most abundant OTUs (KT964893, KT964897 and KT964901) in the core microbiome (Supplementary Table S2) were also found to be present in very low numbers (0.08, 0.22 and 0.06%, respectively) in the surrounding seawater at some locations. While this may indicate that the red coral sheds bacteria in order to regulate its microbial community^34^, it is tempting to speculate that, similar to *Symbiodinium*, these potentially symbiotic microbes have both associated symbiotic stages and free-living stages.

Here, we provide an in-depth analysis of the *Corallium rubrum*-associated microbiome. Overall, we show that the red coral has a highly distinct microbiome composition within the class of Anthozoa, ubiquitously dominated by bacteria from the order Spirochaetales and a relatively unknown family within the order of Oceanospirillales. The high spatial stability of a core microbiome consisting of 12 OTUs suggests strong and specific associations between the red coral and its core resident microbes. Further research is required to elucidate the physiological contributions of these microbial symbionts to host health and the stability of the associations on a temporal scale.

## Methods

### Sample collection

Between April and June 2013, samples of *Corallium rubrum* were collected at 30–40 m depth at five locations: Cap de Creus, Spain (42°18′N 3°18′E), Majorca island, Spain (39°57′N 3°12′E); Cassis, France (43°12′N 5°28′E); Porticcio, Corsica Island, France (41°50′N 8°45′E); Portofino, Italy (44°18′N 9°12′E). Samples were collected from five visually healthy colonies at each location, with the exception of Majorca, where 3 colonies were sampled. Each sample was rinsed twice with 0.2 μm filtered seawater to remove exogenous, loosely associated microorganisms and stored in ice-cold RNA*later* (ThermoFisher Scientific) at 4 °C. At each location, thirty litres of seawater were collected and filtered sequentially through 8 μm, 3 μm and 0.2-μm Whatman Nuclepore Track-Etched filters (Sigma-Aldrich) and the retentate was kept in RNA*later* at 4 °C.

### DNA extraction and 16S rRNA gene amplicon library preparation

Tissues and associated microbes were removed from the skeleton using an airbrush with 5 mL 0.2 M EDTA, and filter retentates were bead beaten prior to DNA extraction. Tissue slurries were centrifuged, and DNA was extracted from the pelleted *C. rubrum* tissues and seawater filter retentate using the Genomic DNA Buffer Set and Genomic-tip 20/G columns (QIAGEN) according to the manufacturer’s sample preparation and lysis protocol for tissues. 16S rDNA amplicon libraries were generated using the 8F/338R primer set^35^, which covers the V1-V2 hypervariable regions of the bacterial 16S rRNA gene, in a 50 μl volume containing 0.2 μM of each primer, 2.5 mM dNTP, 5% *v/v* dimethyl sulfoxide (DMSO), 10 ng template DNA, 1x AccuBuffer and 0.5 μl ACCUZYME DNA polymerase (Bioline GmbH). The PCR protocol consisted of an initial denaturation step of 95 °C for 3 min followed by 15 amplification cycles (denaturation at 98 °C for 10 s, annealing at 55 °C for 10 s and extension at 72 °C for 45 s). Of this reaction, 1 μl was used as a template in a secondary PCR reaction performed under the same conditions, with the exception that primers were replaced by primers designed to overlap the compatible adaptors, barcodes and indexing for Illumina multiplexing sequencing^35^ for 20 cycles and a final extension at 72 °C for 5 min. Final PCRs were dried in a vacuum centrifuge concentrator (SpeedVac, Thermo Scientific), re-suspended in 30 μl of double-distilled water and separated on a 2% agarose gel. PCR products of the correct amplicon size were extracted using the Qiaquick Gel Extraction Kit (QIAGEN). Libraries were sent for paired-end sequencing on the Illumina MiSeq platform (version 3 chemistry – 2 × 300 bp, 600 cycles) at the Helmholtz Centre for Infection Research (Braunschweig, Germany). Image analysis and base calling were performed using the Illumina Pipeline (version 1.7).

### 16S rRNA gene amplicon data analysis

The QIIME pipeline^36^ was used for data processing. MiSeq sequencing produced 3,536,820 reads. Forward and reverse reads were joined using the fast-join method and the resulting .fastq file was used to generate quality (.qual) and reads (.fasta) files for further processing. The split_libraries.py script was used to remove low quality (Phred <25) sequences, reads <200 bp or >500 bp in length, primers and barcodes, and to assign each read to its respective sample. Chimeric sequences were identified (on average 5.6%) against the curated SILVA reference database (version 119)^37^ and removed using UCHIME^38^. Operational Taxonomic Units (OTUs) were defined at the level of 97% similarity followed by taxonomy assignments against the SILVA reference database (version 119) using the UCLUST algorithm^39^. Singletons, unassigned OTUs, and OTUs classified as chloroplast or mitochondria were removed from the dataset. Overall microbiomes (rarefied to 24,509 reads for each sample) as well as the community of locally stable microbial associates (OTUs present in 100% of the samples at each sampling location; rarefied to 23,940 reads for each sample) and the core microbiome (OTUs present in all samples; rarefied to 23,890 reads for each sample) were determined. Alpha diversity metrics (total observed number of OTUs, predicted species (chao1), Shannon-Wiener diversity index, Simpson’s Evenness and species richness estimate^40^) and beta diversity UniFrac distance matrices were generated from OTU tables using the QIIME pipeline. The complete dataset has been deposited in the NCBI Sequence Read Archive (SRA) with accession number SRP071002.

### Statistical Analysis

Differences in alpha diversity metrics among sampling locations were analysed using an Analysis of Variance (ANOVA). To visualise differences in OTU membership between *C. rubrum*-associated bacterial communities, principal coordinate analysis (PCoA) was performed on unweighted UniFrac distance matrices. To visualise differences in microbial community structures based on species abundances, Bray-Curtis dissimilarity matrices were generated from OTU tables and subsequently subjected to PCoA. Homogeneity of multivariate dispersions between locations were tested using Permutational Analysis of Multivariate Dispersions (PERMDISP), and were only found to be significant for unweighted UniFrac distance matrices (p < 0.03). Permutational Analysis of Variance (PERMANOVA) and pair-wise comparisons were performed under Type III partial sums of squares and 9999 permutations under the unrestricted model using Monte Carlo simulations to test for differences among bacterial communities at different locations on the unweighted UniFrac and Bray-Curtis dissimilarity matrices. To determine the OTUs driving the observed differences between red coral-associated microbial communities at different locations, Similarity Percentage (SIMPER) analyses were applied to the OTU tables. All beta diversity analyses were conducted using PRIMER 6 & PERMANOVA+(PRIMER-E Ltd)^41^.

### Phylogenetic analyses of gorgonian associated microbes

The QIIME pipeline^36^ was used to identify the most representative sequence of each Spirochaetales OTU. An initial phylogenetic tree was built based on the published full-length 16S rDNA sequences available from GenBank, using the Maximum Parsimony approach in MEGA6^42^, and the representative sequences from this study were added to build the final tree, based on a 300 bp alignment matrix. The bootstrap consensus tree was inferred from 1,000 replicates. Sequences of core microbiome OTUs were deposited in the GenBank database under the accession numbers KT964892 – KT964903.

## Additional Information

**Accession codes:** The sequences of the core microbiome OTUs found in this study were deposited in the GenBank database under accession numbers KT964898 – KT964901. 16S rRNA amplicon sequence data: NCBI SRP071002. 

**How to cite this article**: van de Water, J. A. J. M. *et al*. Spirochaetes dominate the microbial community associated with the red coral *Corallium rubrum* on a broad geographic scale. *Sci. Rep*. **6**, 27277; doi: 10.1038/srep27277 (2016).

## Supplementary Material

Supplementary Information

## Figures and Tables

**Table 1:** Overview of alpha diversity metrics (average ± s.d.) for each sampling location.

Location	Species Richness	Simpson Evenness	Chao1 estimate	Shannon-Wiener Index	OTUs in microbiome	Locally stable microbial associates
Cap de Creus	352 ± 31	0.010 ± 0.003	554 ± 69	1.93 ± 0.27	234 ± 27	29
Cassis	350 ± 31	0.013 ± 0.002	547 ± 91	2.29 ± 0.20	236 ± 16	45
Majorca	376 ± 39	0.013 ± 0.001	479 ± 115	2.37 ± 0.35	272 ± 25	68
Porticcio	359 ± 28	0.011 ± 0.002	584 ± 77	2.06 ± 0.27	236 ± 15	41
Portofino	422 ± 34	0.008 ± 0.003	693 ± 99	1.63 ± 0.32	272 ± 37	34
Seawater	1058 ± 177	0.016 ± 0.005	1189 ± 197	5.98 ± 0.37	854 ± 147	

## References

[b1] Bramanti L. . Detrimental effects of ocean acidification on the economically important Mediterranean red coral (*Corallium rubrum*). Glob Change Biol 19, 1897–1908 (2013). 10.1111/gcb.12171 23505003

[b2] Cerrano C. . A catastrophic mass-mortality episode of gorgonians and other organisms in the Ligurian Sea (North-western Mediterranean), summer 1999. Ecol Lett 3, 284–293 (2000).

[b3] Bourne D., Iida Y., Uthicke S. & Smith-Keune C. Changes in coral-associated microbial communities during a bleaching event. Isme J 2, 350–363 (2008). 18059490 10.1038/ismej.2007.112

[b4] Littman R., Willis B. L. & Bourne D. G. Metagenomic analysis of the coral holobiont during a natural bleaching event on the Great Barrier Reef. Environ Microbiol Rep 3, 651–660 (2011). 23761353 10.1111/j.1758-2229.2010.00234.x

[b5] Bramanti L., Magagnini G., De Maio L. & Santangelo G. Recruitment, early survival and growth of the Mediterranean red coral *Corallium rubrum* (L 1758), a 4-year study. J Exp Mar Biol Ecol 314, 69–78 (2005).

[b6] La Rivière M., Garrabou J. & Bally M. Evidence for host specificity among dominant bacterial symbionts in temperate gorgonian corals. Coral Reefs, 1–12 (2015).

[b7] Pasquale V. . Cultivable heterotrophic bacteria associated to *Corallium rubrum*. Biol Mar Medit 18, 274–275 (2011).

[b8] Vezzulli L., Pezzati E., Huete-Stauffer C., Pruzzo C. & Cerrano C. 16SrDNA Pyrosequencing of the Mediterranean Gorgonian Paramuricea clavata Reveals a Link among Alterations in Bacterial Holobiont Members, Anthropogenic Influence and Disease Outbreaks. PLoS ONE 8, e67745 (2013). 23840768 10.1371/journal.pone.0067745 PMC3694090

[b9] La Rivière M., Roumagnac M., Garrabou J. & Bally M. Transient Shifts in Bacterial Communities Associated with the Temperate Gorgonian *Paramuricea clavata* in the Northwestern Mediterranean Sea. PLoS ONE 8, e57385 (2013). 23437379 10.1371/journal.pone.0057385 PMC3577713

[b10] Ransome E., Rowley S. J., Thomas S., Tait K. & Munn C. B. Disturbance to conserved bacterial communities in the cold-water gorgonian coral *Eunicella verrucosa*. FEMS Microbiol Ecol 90, 404–416 (2014). 25078065 10.1111/1574-6941.12398

[b11] Bayer T. . Bacteria of the genus *Endozoicomonas* dominate the microbiome of the Mediterranean gorgonian coral *Eunicella cavolini*. Mar Ecol-Prog Ser 479, 75–84 (2013).

[b12] Bayer T. . The microbiome of the Red Sea coral *Stylophora pistillata* is dominated by tissue-associated *Endozoicomonas* bacteria. Appl. Environ. Microbiol. (2013). 10.1128/AEM.00695-13 PMC3719505 23709513

[b13] Lema K. A., Willis B. L. & Bourne D. G. Amplicon pyrosequencing reveals spatial and temporal consistency in diazotroph assemblages of the *Acropora millepora* microbiome. Environ Microbiol 16, 3345–3359 (2014). 24373029 10.1111/1462-2920.12366

[b14] Morrow K. M., Moss A. G., Chadwick N. E. & Liles M. R. Bacterial Associates of Two Caribbean Coral Species Reveal Species-Specific Distribution and Geographic Variability. Appl. Environ. Microbiol. 78, 6438–6449 (2012). 22773636 10.1128/AEM.01162-12 PMC3426691

[b15] Dishaw L. J. . The Gut of Geographically Disparate *Ciona intestinalis* Harbors a Core Microbiota. PLoS ONE 9, e93386 (2014). 24695540 10.1371/journal.pone.0093386 PMC3973685

[b16] Jensen S., Duperron S., Birkeland N.-K. & Hovland M. Intracellular Oceanospirillales bacteria inhabit gills of *Acesta bivalves*. FEMS Microbiol Ecol 74, 523–533 (2010). 21044098 10.1111/j.1574-6941.2010.00981.x

[b17] Raina J.-B., Tapiolas D., Willis B. L. & Bourne D. G. Coral-Associated Bacteria and Their Role in the Biogeochemical Cycling of Sulfur. Appl. Environ. Microbiol. 75, 3492–3501 (2009). 19346350 10.1128/AEM.02567-08 PMC2687302

[b18] Roder C., Bayer T., Aranda M., Kruse M. & Voolstra C. R. Microbiome structure of the fungid coral *Ctenactis echinata* aligns with environmental differences. Mol Ecol 24, 3501–3511 (2015). 26018191 10.1111/mec.13251 PMC4736464

[b19] Ruehland C. . Multiple bacterial symbionts in two species of co-occurring gutless oligochaete worms from Mediterranean sea grass sediments. Environ Microbiol 10, 3404–3416 (2008). 18764872 10.1111/j.1462-2920.2008.01728.x

[b20] Brune A. Symbiotic digestion of lignocellulose in termite guts. Nat Rev Micro 12, 168–180 (2014). 10.1038/nrmicro3182 24487819

[b21] Mayasich S. A. & Smucker R. A. Role of *Cristispira* sp. and other bacteria in the chitinase and chitobiase activities of the crystalline style of *Crassostrea virginica* (Gmelin). Microb Ecol 14, 157–166 (1987). 24202643 10.1007/BF02013020

[b22] Lilburn T. G. . Nitrogen Fixation by Symbiotic and Free-Living Spirochetes. Science 292, 2495–2498 (2001). 11431569 10.1126/science.1060281

[b23] Closek C. J. . Coral transcriptome and bacterial community profiles reveal distinct Yellow Band Disease states in *Orbicella faveolata*. Isme J 8, 2411–2422 (2014). 24950107 10.1038/ismej.2014.85 PMC4260706

[b24] Frias-Lopez J., Zerkle A. L., Bonheyo G. T. & Fouke B. W. Partitioning of Bacterial Communities between Seawater and Healthy, Black Band Diseased, and Dead Coral Surfaces. Appl. Environ. Microbiol. 68, 2214–2228 (2002). 11976091 10.1128/AEM.68.5.2214-2228.2002 PMC127591

[b25] Sekar R., Kaczmarsky L. T. & Richardson L. L. Microbial community composition of black band disease on the coral host *Siderastrea siderea* from three regions of the wider Caribbean. Mar Ecol-Prog Ser 362, 85–98 (2008).

[b26] Ainsworth T. D. . The coral core microbiome identifies rare bacterial taxa as ubiquitous endosymbionts. Isme J 9, 2261–2274 (2015). 25885563 10.1038/ismej.2015.39 PMC4579478

[b27] Ng J. C.-Y. . Pyrosequencing of the bacteria associated with *Platygyra carnosus* corals with skeletal growth anomalies reveals differences in bacterial community composition in apparently healthy and diseased tissues. Front Microbiol 6 (2015). 10.3389/fmicb.2015.01142 PMC4611154 26539174

[b28] Kellogg C. A., Lisle J. T. & Galkiewicz J. P. Culture-Independent Characterization of Bacterial Communities Associated with the Cold-Water Coral *Lophelia pertusa* in the Northeastern Gulf of Mexico. Appl. Environ. Microbiol. 75, 2294–2303 (2009). 19233949 10.1128/AEM.02357-08 PMC2675238

[b29] Penn K., Wu D., Eisen J. A. & Ward N. Characterization of Bacterial Communities Associated with Deep-Sea Corals on Gulf of Alaska Seamounts. Appl. Environ. Microbiol. 72, 1680–1683 (2006). 16461727 10.1128/AEM.72.2.1680-1683.2006 PMC1392894

[b30] Gray M. A., Stone R. P., McLaughlin M. R. & Kellogg C. A. Microbial consortia of gorgonian corals from the Aleutian islands. FEMS Microbiol Ecol 76, 109–120 (2011). 21223327 10.1111/j.1574-6941.2010.01033.x

[b31] Sunagawa S. . Bacterial diversity and White Plague Disease-associated community changes in the Caribbean coral *Montastraea faveolata*. Isme J 3, 512–521 (2009). 19129866 10.1038/ismej.2008.131

[b32] Leadbetter J. R., Schmidt T. M., Graber J. R. & Breznak J. A. Acetogenesis from H_2_ Plus CO_2_ by Spirochetes from Termite Guts. Science 283, 686–689 (1999). 9924028 10.1126/science.283.5402.686

[b33] Sharp K. H., Distel D. & Paul V. J. Diversity and dynamics of bacterial communities in early life stages of the Caribbean coral Porites astreoides. Isme J 6, 790–801 (2012). 22113375 10.1038/ismej.2011.144 PMC3309355

[b34] Garren M. & Azam F. Corals shed bacteria as a potential mechanism of resilience to organic matter enrichment. Isme J 6, 1159–1165 (2012). 22189494 10.1038/ismej.2011.180 PMC3358026

[b35] Camarinha-Silva A. . Comparing the anterior nare bacterial community of two discrete human populations using Illumina amplicon sequencing. Environ Microbiol 16, 2939–2952 (2014). 24354520 10.1111/1462-2920.12362

[b36] Caporaso J. G. . QIIME allows analysis of high-throughput community sequencing data. Nat Methods 7, 335–336 (2010). 20383131 10.1038/nmeth.f.303 PMC3156573

[b37] Quast C. . The SILVA ribosomal RNA gene database project: improved data processing and web-based tools. Nucleic Acids Res 41, D590–D596 (2013). 23193283 10.1093/nar/gks1219 PMC3531112

[b38] Edgar R. C., Haas B. J., Clemente J. C., Quince C. & Knight R. UCHIME improves sensitivity and speed of chimera detection. Bioinformatics 27, 2194–2200 (2011). 21700674 10.1093/bioinformatics/btr381 PMC3150044

[b39] Edgar R. C. Search and clustering orders of magnitude faster than BLAST. Bioinformatics 26, 2460–2461 (2010). 20709691 10.1093/bioinformatics/btq461

[b40] Colwell R. K. . Models and estimators linking individual-based and sample-based rarefaction, extrapolation and comparison of assemblages. J Plant Ecol 5, 3–21 (2012).

[b41] Clarke K. R. & Warwick R. M. Change in marine communities: an approach to statistical analysis and interpretation 2nd edition PRIMER-E, Plymouth, 172pp. 172 (2001).

[b42] Tamura K., Stecher G., Peterson D., Filipski A. & Kumar S. MEGA6: Molecular Evolutionary Genetics Analysis Version 6.0. Mol Biol Evol 30, 2725–2729 (2013). 24132122 10.1093/molbev/mst197 PMC3840312

