# Supplementary material for: Spirochaetes dominate the microbial community associated with the red coral Corallium rubrum on a broad geographic scale
Source: Sci Rep. 2016 Jun 6;6:27277. doi: 10.1038/srep27277 (PMC4893704; doi:10.1038/srep27277)
Supplement: Supplementary Information [file srep27277-s1.pdf]

Supplementary Figures & Tables

for

Spirochaetes **dominate the microbial community associated with the red coral** ***Corallium rubrum*** **on a broad geographical scale**

Jeroen A.J.M. van de Water, Rémy Melkonian, Howard Junca, Christian R. Voolstra, Stéphanie Reynaud, Denis Allemand, Christine Ferrier-Pagès

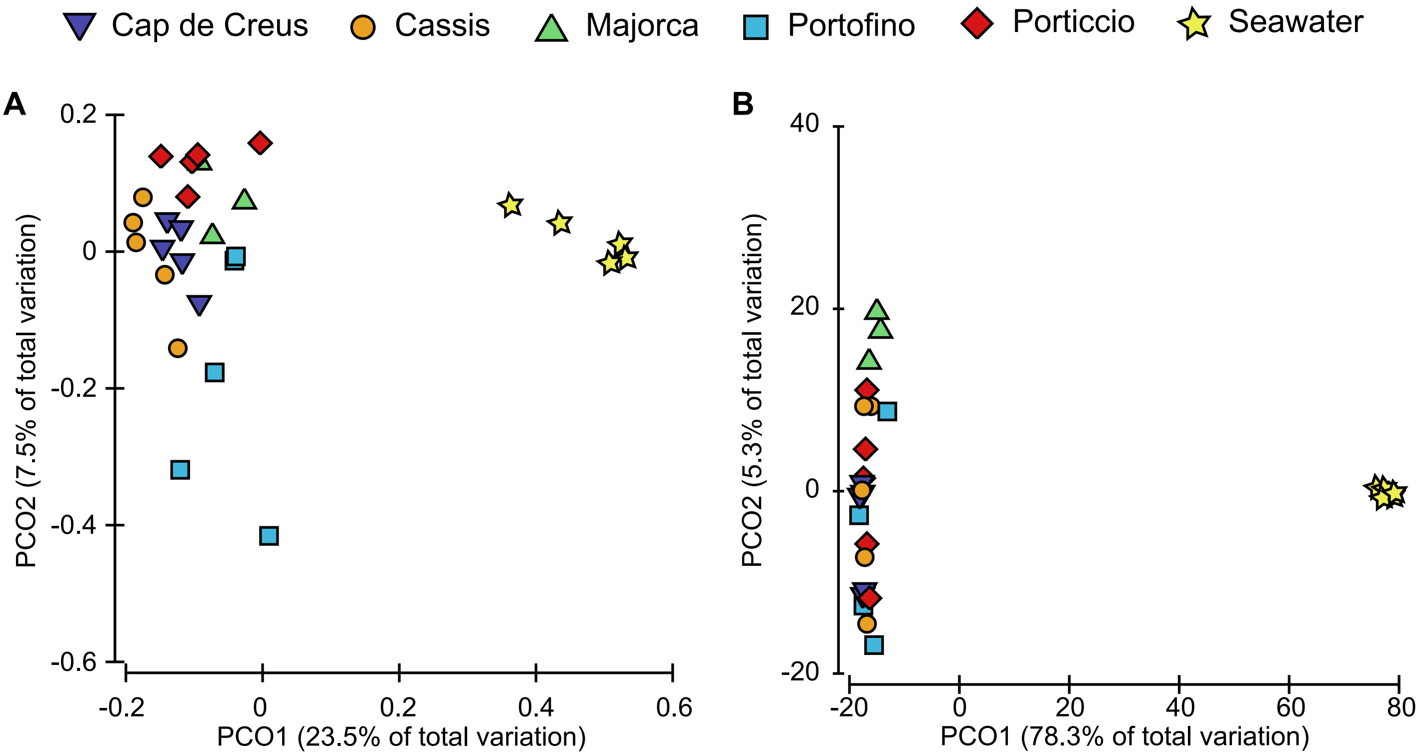

C

Source	df	SS	MS	Pseudo-F	P(perm)	Unique perms	P(Monte Carlo)
C.r. vs SW	1	1.3753	1.3753	7.477	0.0001	9354	***0.0001***
Residuals	26	4.7822	0.18393				
Total	27	6.1575					

D

Source	df	SS	MS	Pseudo-F	P(perm)	Unique perms	P(Monte Carlo)
C.r. vs SW	1	36616	36616	93.487	0.0001	9396	***0.0001***
Residuals	26	10184	391.67				
Total	27	46800					

Supplementary Figure S1 - Beta diversity comparison between seawater and *Corallium rubrum* samples. Principal coordinate analysis based on (A) unweighted UniFrac distances and (B) Bray-Curtis dissimilarity matrix to show differences in membership and community structure, respectively. Statistical results of a Permutational Analysis of Variance to test for differences in (C) membership and (D) community structure between seawater (SW) and *Corallium rubrum* (C.r.) samples based on Monte Carlo simulations.

A

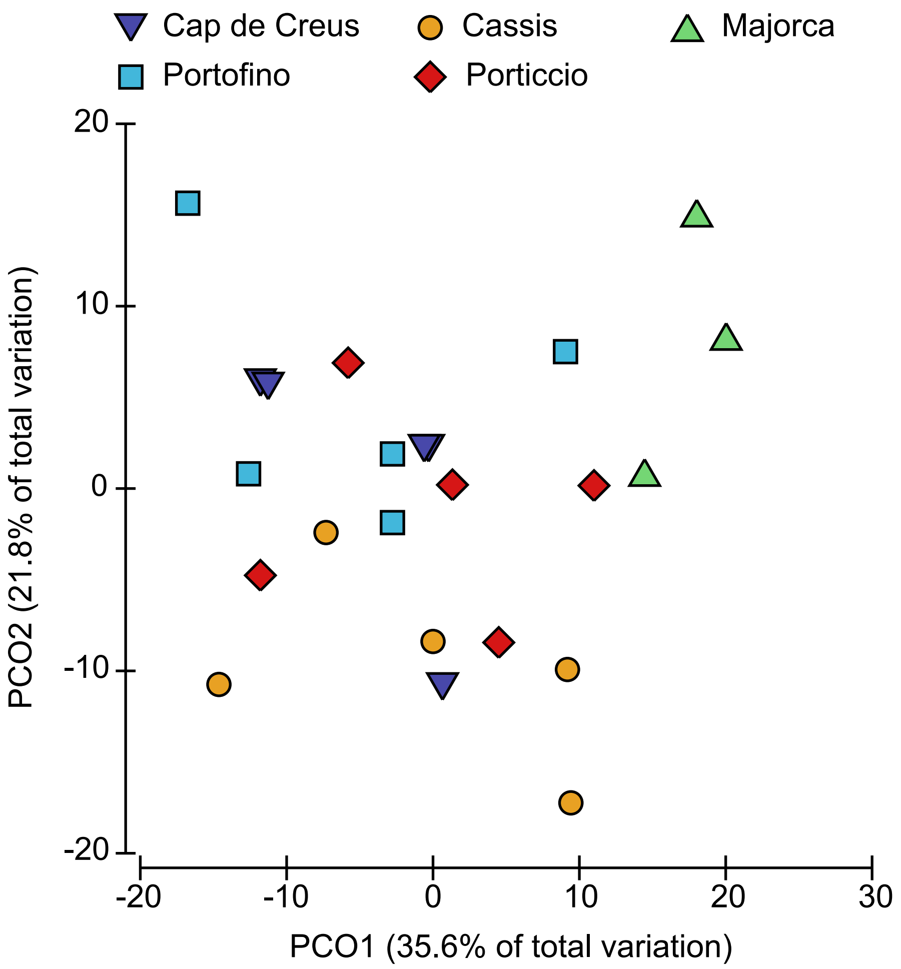

B

Source	df	SS	MS	Pseudo-F	P(perm)	Unique permutations	P(Monte Carlo)
Location	4	2645.2	661.3	2.7391	0.0001	9891	***0.0015***
Residuals	18	4345.7	241.43				
Total	22	6990.9					

Groups	t	P(perm)	Unique perms	P(Monte Carlo)
Cap de Creus, Portofino	0.90113	0.6115	126	0.4834
Cap de Creus, Porticcio	1.1148	0.2981	126	0.2968
Cap de Creus, Cassis	1.4142	0.1099	126	0.1290
Cap de Creus, Majorca	2.5878	0.0156	56	***0.0045***
Portofino, Porticcio	1.1652	0.1888	126	0.2719
Portofino, Cassis	1.5559	0.0472	126	0.0906
Portofino, Majorca	2.0268	0.0166	56	***0.0239***
Porticcio, Cassis	1.3046	0.1656	126	0.1660
Porticcio, Majorca	1.9817	0.0188	56	***0.0233***
Cassis, Majorca	2.3886	0.0176	56	***0.0146***

Supplementary Figure S2 - Spatial differences in the overall bacterial community of *Corallium rubrum*. (A) Principal coordinate analysis based on Bray-Curtis dissimilarity matrix and (B) outcomes of the Permutational Analysis of Variance to test for differences between the different sampling locations based on Monte Carlo simulations.

A

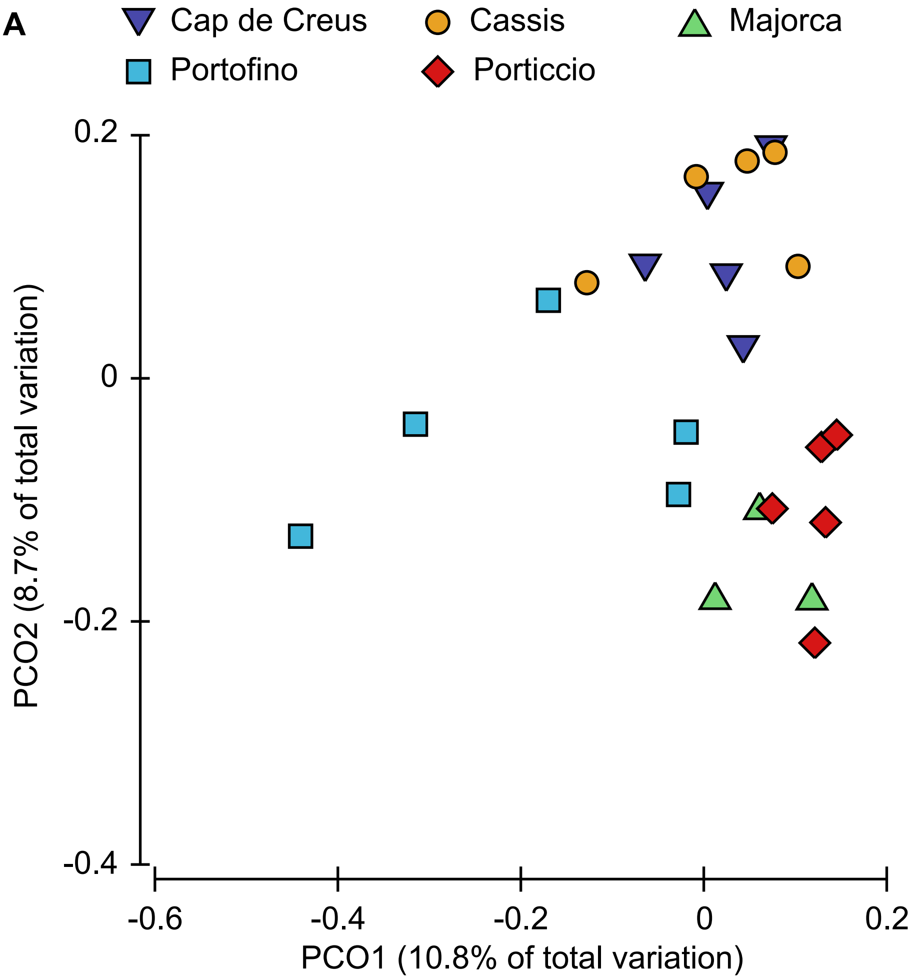

B

Source	df	SS	MS	Pseudo-F	P(perm)	Unique permutations	P(Monte Carlo)
Location	4	1.1522	0.28805	1.6556	0.0001	9637	***0.0025***
Residuals	18	3.1318	0.17399				
Total	22	4.2840					

Groups	t	P(perm)	Unique perms	P(Monte Carlo)
Cap de Creus, Portofino	1.2167	0.0067	126	0.1789
Cap de Creus, Porticcio	1.3294	0.0092	126	0.0913
Cap de Creus, Cassis	1.1184	0.0072	126	0.2814
Cap de Creus, Majorca	1.2811	0.0181	56	0.1582
Portofino, Porticcio	1.4117	0.0087	126	0.068
Portofino, Cassis	1.3039	0.007	126	0.1189
Portofino, Majorca	1.2324	0.0342	56	0.1987
Porticcio, Cassis	1.3571	0.009	126	0.0853
Porticcio, Majorca	1.2598	0.0167	56	0.1738
Cassis, Majorca	1.3364	0.016	56	0.1227

Supplementary Figure S3 - Spatial differences in the membership of the overall bacterial community associated with *Corallium rubrum*. (A) Principal coordinate analysis based on unweighted UniFrac distances and (B) outcomes of the Permutational Analysis of Variance to test for differences between the sampling locations based on Monte Carlo simulations.

A

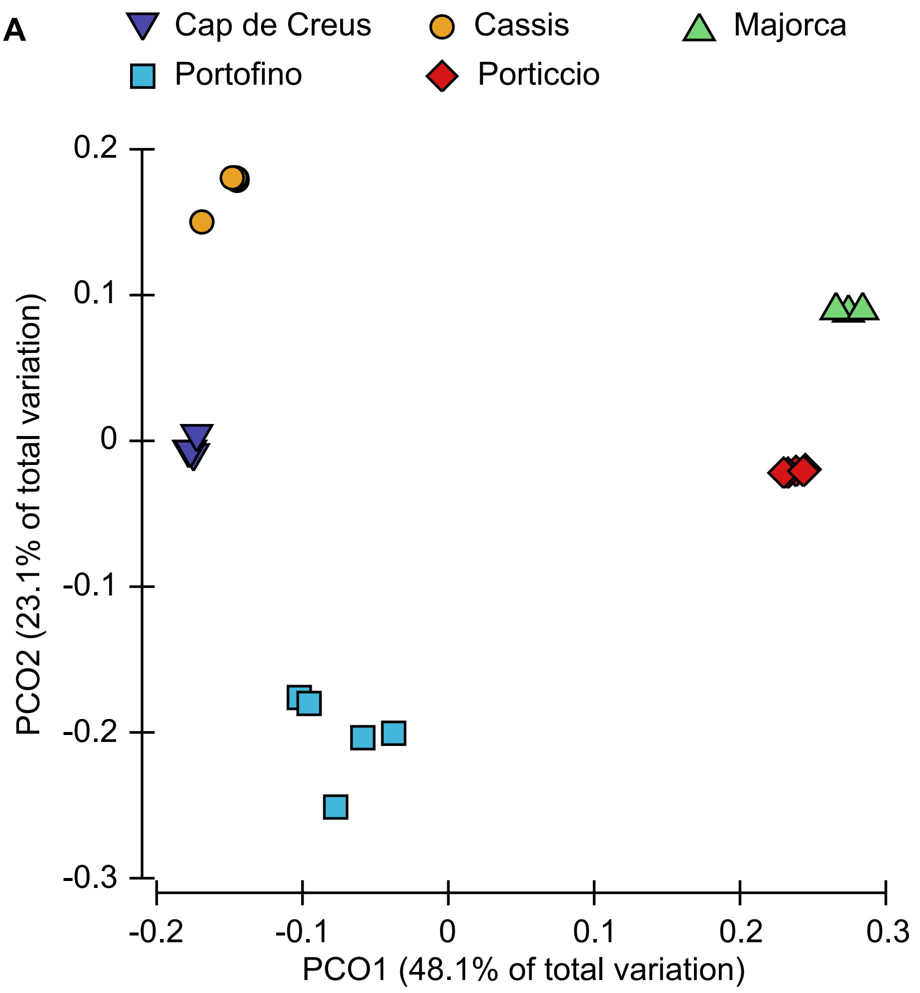

B

Source	df	SS	MS	Pseudo-F	P(perm)	Unique permutations	P(Monte Carlo)
Location	4	1.6348	0.40871	172.18	0.0001	9920	***0.0001***
Residuals	18	4.2E-2	2.37E-3				
Total	22	1.6776					

Groups	t	P(perm)	Unique perms	P(Monte Carlo)
Cap de Creus, Cassis	22.657	0.0081	91	***0.0001***
Cap de Creus, Majorca	28.776	0.017	41	***0.0001***
Cap de Creus, Porticcio	35.5	0.0079	66	***0.0001***
Cap de Creus, Portofino	8.3534	0.009	91	***0.0001***
Cassis, Majorca	25.944	0.0179	56	***0.0001***
Cassis, Porticcio	32.481	0.007	91	***0.0001***
Cassis, Portofino	8.9281	0.0076	126	***0.0001***
Majorca, Portofino	19.747	0.0185	41	***0.0001***
Majorca, Portofino	8.6506	0.0173	56	***0.0001***
Porticcio, Portofino	9.4549	0.0083	91	***0.0001***

Supplementary Figure S4 - Spatial differences in the community membership of locally stable microbial associates of *Corallium rubrum*. (A) Principal coordinate analysis based on unweighted UniFrac distances and (B) outcomes of the Permutational Analysis of Variance to test for differences between the sampling locations based on Monte Carlo simulations.

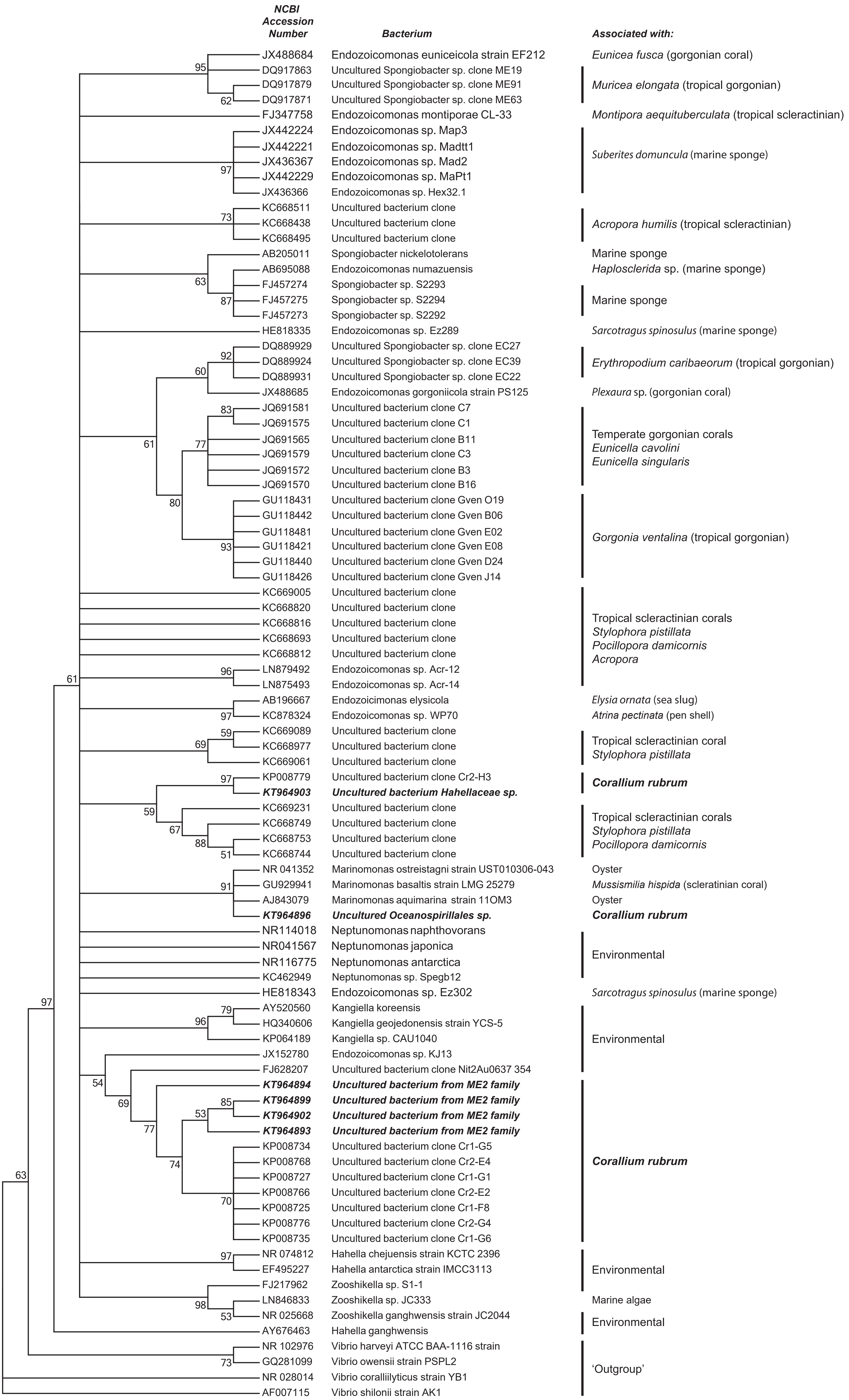

Suplementary Figure S5: Oceanospirillales phylogeny. A 16S ribosomal RNA reconstruction of the Order Oceanospirillales (class gamma-proteobacteria) created based on the maximum parsimony model. Percentages of 1000 bootstrap replicates are indicated next to the tree nodes if they are > 50%. Sequences from the present study are in **bold** and *italic*. The tree is rooted using sequences from the genus *Vibrio* (order of Vibrionales; class gamma-proteobacteria) as the outgroup.

Supplementary Table S1 - Overview of the contribution of the core microbiome to the microbial community associated with Corallium rubrum. Numbers listed are number of reads in the (core) microbiome per sample. Contribution of the core microbiome to the overall microbiome is presented in percentages (%).

	Overall microbiome	Core microbiome all samples	Contribution core microbiome	
Cap de Creus	37126	35996	96.96%	
	33875	32108	94.78%	
	31684	29023	91.60%	
	24509	23890	97.47%	
	34268	32827	95.79%	
Cassis	39164	35562	90.80%	
	30338	28122	92.70%	
	31687	29538	93.22%	
	33891	31529	93.03%	
	30944	29139	94.17%	
Majorca	38975	36488	93.62%	
	25956	24655	94.99%	
	31593	30504	96.55%	
Porticcio	44197	43148	97.63%	
	35890	32335	90.09%	
	32665	29293	89.68%	
	42728	39580	92.63%	
	36548	34384	94.08%	
Portofino	30421	29902	98.29%	
	386862	378170	97.75%	
	466229	454649	97.52%	
	47458	46069	97.07%	
	49280	31797	64.52%	*Outlier
Average overall			94.57%	
SD			2.65%	

	Average per location	Standard Deviation
Cap de Creus	95.32%	1.04%
Cassis	92.78%	0.55%
Majorca	95.05%	0.85%
Porticcio	92.82%	1.45%
Portofino	97.66%	0.23%

Table S2 - Taxonomy assignment and abundance of the core microbiome members of *Corallium rubrum*

Phylum	Class	Order	Family	NCBI Accession Number	OTU#	Abundance	Standard Deviation
Candidate division OD1				KT964898	denovo6421	1.64%	1.51%
Proteobacteria	Gammaproteobacteria	Oceanospirillales	Hahellaceae	KT964903	denovo13612	3.43%	5.49%
Proteobacteria	Gammaproteobacteria	Oceanospirillales	ME2	KT964893	denovo1837	20.09%	6.94%
Proteobacteria	Gammaproteobacteria	Oceanospirillales	ME2	KT964902	denovo11449	0.08%	0.05%
Proteobacteria	Gammaproteobacteria	Oceanospirillales	ME2	KT964894	denovo3318	0.07%	0.04%
Proteobacteria	Gammaproteobacteria	Oceanospirillales	ME2	KT964899	denovo6965	0.02%	0.01%
Proteobacteria	Gammaproteobacteria	Oceanospirillales		KT964896	denovo5353	2.05%	2.59%
Spirochaetae	Spirochaetes	Spirochaetales	Leptospiraceae	KT964895	denovo5318	0.24%	0.31%
Spirochaetae	Spirochaetes	Spirochaetales	Spirochaetaceae	KT964897	denovo5472	59.87%	10.17%
Spirochaetae	Spirochaetes	Spirochaetales	Spirochaetaceae	KT964892	denovo1514	0.01%	0.01%
Spirochaetae	Spirochaetes	Spirochaetales	Spirochaetaceae	KT964900	denovo7341	0.04%	0.01%
Spirochaetae	Spirochaetes	Spirochaetales	Spirochaetaceae	KT964901	denovo10781	12.45%	7.36%

Table S3 - Similarity Percentage (SIMPER) analysis results indicating the main OTUs driving the differences in microbial communities associated with *Corallium rubrum* between Majorca and the other sampling locations. Results indicated explain >80% of the dissimilarity observed.

Cap de Creus & Majorca
Average dissimilarity = 29.66

Taxon	NCBI Accession Number	**Cap de Creus** Average Abundance	**Majorca** Average Abundance	Average Dissimilarity	Standard deviation	Contribution (%)	Cumulative (%)
Spirochaetaceae	KT964897	14987	10980.67	8.17	2.04	27.55	27.55
Hahellaceae	KT964903	173.8	3635	7.06	2.41	23.8	51.36
Spirochaetaceae	KT964901	2777.8	4992.67	4.85	1.2	16.36	67.72
Oceanospir. ME2	KT964893	4281.8	3120.67	3.43	1.14	11.57	79.28
Oceanospirillales	KT964896	490.6	14	0.98	1.45	3.29	82.57

Portofino & Majorca
Average dissimilarity = 32.87

Taxon	NCBI Accession Number	**Portofino** Average Abundance	**Majorca** Average Abundance	Average Dissimilarity	Standard deviation	Contribution (%)	Cumulative (%)
Spirochaetaceae	KT964897	15242.2	10980.67	9.21	1.57	28.01	28.01
Hahellaceae	KT964903	312.8	3635	6.78	2.31	20.62	48.63
Spirochaetaceae	KT964901	2356	4992.67	5.6	1.31	17.05	65.68
Oceanospir. ME2	KT964893	3982	3120.67	3.35	1.39	10.2	75.88
Oceanospirillales	KT964896	298	14	0.58	2.7	1.76	77.64
Candidate div. OD1	KT964898	63.6	298.33	0.48	1.86	1.46	79.1
Leptospiraceae	KT964895	10.8	218.67	0.42	2.98	1.29	80.39

Porticcio & Majorca
Average dissimilarity = 27.47

Taxon	NCBI Accession Number	**Porticcio** Average Abundance	**Majorca** Average Abundance	Average Dissimilarity	Standard deviation	Contribution (%)	Cumulative (%)
Hahellaceae	KT964903	524	3635	6.35	2.11	23.1	23.1
Spirochaetaceae	KT964897	13689.6	10980.67	5.53	1.54	20.12	43.22
Spirochaetaceae	KT964901	3053.8	4992.67	5.46	1.26	19.86	63.08
Oceanospir. ME2	KT964893	5033.8	3120.67	4.37	1.43	15.9	78.98
Rickettsialis	not core	265.2	5.33	0.55	0.49	1.98	80.96

Cassis & Majorca
Average dissimilarity = 31.64

Taxon	NCBI Accession Number	**Cassis** Average Abundance	**Majorca** Average Abundance	Average Dissimilarity	Standard deviation	Contribution (%)	Cumulative (%)
Hahellaceae	KT964903	430.6	3635	6.54	2.18	20.66	20.66
Spirochaetaceae	KT964901	1949	4992.67	6.39	1.36	20.2	40.87
Oceanospir. ME2	KT964893	5901.4	3120.67	5.74	1.52	18.15	59.02
Spirochaetaceae	KT964897	12462.8	10980.67	4.22	1.43	13.35	72.37
Oceanospirillales	KT964896	1311.2	14	2.65	2.03	8.37	80.74

Table S4 - Identity similarity of *Corallium rubrum* core microbiome members with its closest sequenced relatives and closest cultured relative, based on 300 bp 16S amplicon

		**Closest Sequenced Relative**				**Closest Cultured Relative**			
**NCBI Accession Number**	**OTU#**	**NCBI Accession Number**	**% Identity**	**E-value**	**Source**	**NCBI Accession Number**	**% Identity**	**E-value**	**Bacterium**
KT964898	denovo6421	JQ214381	92%	3.00E-115	*Tursiops truncatus*	CP011215	86%	2.00E-91	Parcubacteria GW2011_OD1_34_28
KT964903	denovo13612	KP008779	100%	5.00E-157	*Corallium rubrum*	AB205011	95%	2.00E-143	*Spongiobacter nickelotolerans*
KT964893	denovo1837	KP008779	99%	8.00E-155	*Corallium rubrum*	KP064189	87%	6.00E-104	*Kangiella* sp. CAU1040
KT964902	denovo11449	KP008776	99%	2.00E-150	*Corallium rubrum*	KC462949	88%	3.00E-96	*Neptunomonas* sp. Spegb12
KT964894	denovo3318	KP008776	96%	3.00E-141	*Corallium rubrum*	KP064189	88%	1.00E-105	*Kangiella* sp. CAU1040
KT964899	denovo6965	KP008776	98%	1.00E-146	*Corallium rubrum*	KP064189	86%	7.00E-93	*Kangiella* sp. CAU1040
KT964896	denovo5353	FJ628207	94%	1.00E-145	Environmental	NR041352	92%	2.00E-125	*Marinomonas ostreistagni*
KT964895	denovo5318	FJ203546	91%	6.00E-118	*Montastrea faveolata*	AY996789	76%	2.00E-49	*Leptospira fainei*
KT964897	denovo5472	KP008742	100%	3.00E-154	*Corallium rubrum*	FJ380060	79%	2.00E-67	*Spirochaeta* sp. SR
KT964892	denovo1514	DQ395500	97%	7.00E-156	*Isidella tentaculum*	FJ380060	78%	5.00E-62	*Spirochaeta* sp. SR
KT964900	denovo7341	KP008742	99%	7.00E-156	*Corallium rubrum*	FJ380060	77%	1.00E-57	*Spirochaeta* sp. SR
KT964901	denovo10781	KP008759	100%	3.00E-153	*Corallium rubrum*	FJ380060	86%	1.00E-102	*Spirochaeta* sp. SR
